# Vitamin C mesotherapy versus diode laser for the esthetic management of physiologic gingival hyperpigmentation: a randomized clinical trial

**DOI:** 10.1186/s12903-023-03614-7

**Published:** 2023-11-21

**Authors:** Sara A. Esmat, Naguiba M. El-Sayed, Rania A. Fahmy

**Affiliations:** Department of Oral Medicine, Periodontology, Oral Diagnosis and Oral Radiology, Faculty of Dentistry, Alexandria University, Alexandria, Egypt

**Keywords:** Ascorbic acid, Vitamin C, Diode laser, Gingival depigmentation, Mesotherapy, Melanin, Hyperpigmentation, Smile design, Pink esthetics

## Abstract

**Background:**

Physiologic gingival hyperpigmentation is a common esthetic concern that affects individuals of various ethnicities, and can have a significant impact on individual’s self-confidence and overall quality of life. Thus, this study aimed to clinically assess the effectiveness of intra-mucosal injection of vitamin C versus 980 nm diode laser for the management of physiologic gingival hyperpigmentation.

**Methods:**

Twenty-six healthy non-smoker individuals with physiologic gingival hyperpigmentation were randomly assigned to two groups. Group I received intra-mucosal injection of vitamin C (L-Ascorbic acid 1000 mg/5 ml), and group II was managed using diode laser (980 nm, 1.5 W, continuous wave mode). Clinical evaluation of pigmentation intensity and distribution was performed preoperatively, and at 1, 2 and 3 months postoperatively using two different color assessment indices; Dummett-Gupta Oral Pigmentation Index (DOPI), and Gingival Pigmentation Index (GPI). Additionally, the study assessed pain intensity and patients’ satisfaction.

**Results:**

Pigmentation scores decreased significantly between pre-operative visit and different follow-up visits for both treatment modalities (p < 0.0001*). When compared to the vitamin C mesotherapy group, the laser group demonstrated significantly lower gingival pigmentation scores (p < 0.0001*). However, both treatment modalities were equally satisfying for the patients.

**Conclusions:**

Vitamin C mesotherapy and diode laser are both effective in the management of physiologic gingival hyperpigmentation. While diode laser yields better and earlier results, vitamin C mesotherapy offers a cost-effective, safe and minimally invasive approach that is equally satisfying for the patients seeking esthetic enhancements.

**Trial registration:**

The study was registered on ClinicalTrials.gov (NCT05608057) on (01/11/2022).

## Background

In today’s world, patients’ esthetic demands and expectations have greatly risen, with all current trends focusing on facial esthetics and smile design. A smile plays a vital role in non-verbal communication, and greatly influences an individual’s self-esteem. It holds significant value in social interactions impacting relationships, professional image, and personality development. Studies have consistently shown that individuals who are satisfied with their physical appearance, particularly their smile, tend to be more confident and successful. Conversely, a compromised smile negatively impacts personal image and overall quality of life [1, 2].

The role of pink esthetics in smile design cannot be overlooked, as the harmony of a smile is not solely determined by the shade, shape and arrangement of teeth, but also by the appealing and healthy appearance of the surrounding gingival tissues [3].

Healthy gingival color is generally described as coral pink [4]. The pigments present within the gingival tissue such as melanin, carotene, oxyhemoglobin and reduced hemoglobin play a significant role in determining the inherent color of the gingiva. Among these pigments, melanin exhibits the highest incidence rate [5].

Gingival hyperpigmentation is defined as increased intensity of gingival color through excessive melanin deposition by the melanocytes found within the basal and supra-basal cell layers of the gingival epithelium [6, 7]. The most common cause of gingival hyperpigmentation is physiologic pigmentation, which is described as the most prevalent multifocal or diffuse oral mucosal pigmentation, predominantly found in dark-skin populations [7, 8].

A wide range of depigmentation techniques have been proposed with the surgical approaches being the most widely employed such as conventional scalpel surgery, bur abrasion, electrosurgery, cryosurgery, radiosurgery & more recently, the application of lasers of different types & wavelengths [9, 10].

Laser-tissue interaction is dependent on the laser light’s affinity for specific chromophores found within the tissue [11]. Diode lasers used in dentistry are highly absorbed by soft tissue, and exhibit strong affinity for chromophores like melanin and hemoglobin [10].

Efficient coagulation, short treatment line, accelerated healing, and excellent esthetic outcomes are laser’s known advantages. However, the expensive and sophisticated equipment limits its use, and significantly raises the cost of treatment [12]. Moreover, it requires additional training and education as every dental professional should undergo a comprehensive training program from a reliable provider to ensure proper skill acquisition and proficiency in laser use. It is also important to acknowledge the inherent variability in the learning curve among individuals, highlighting the unique and diverse challenges they may encounter during the learning process. All these limitations represent the overriding obstacles that hinder the implementation of laser technology in dental practice [13].

Vitamin C, also known as Ascorbic acid, has been introduced as a promising therapeutic agent for reducing melanin hyperpigmentation due to its ability to suppress the activity of tyrosinase, the rate-limiting enzyme in melanin biosynthesis [14] Moreover, vitamin C offers additional advantages including antioxidative properties, promotion of collagen synthesis, and immune system support [15].

In the field of dermatology, vitamin C has gained significant popularity and has been widely employed as a safe depigmenting agent. It has shown promising results in the treatment of various dermatological problems associated with hyperpigmentation [16, 17].

However, a recent systematic review indicated that studies investigating the application of vitamin C for gingival depigmentation are scarce, with the majority being experimental studies [17–19]. Only a few clinical studies have used Vitamin C as a sole therapeutic modality for gingival depigmentation, either by topical application or oral mesotherapy technique, and their outcomes were promising [14, 15, 20–22].

The aim behind introducing vitamin C mesotherapy in clinical practice was to develop a novel approach that enables targeted and localized delivery of the therapeutic agent, vitamin C, to the site of concern, maximizing its efficacy and ensuring a more potent and focused effect. On the financial aspect, vitamin C mesotherapy stands out as a cost-effective treatment modality, allowing successful drug delivery using inexpensive equipment, and requiring short learning curve for the general practitioner [23].

To the best of our knowledge, no clinical trial has exclusively compared the clinical effect of vitamin C mesotherapy with laser ablation therapy for gingival depigmentation. Therefore, the aim of this study was to compare the effectiveness of vitamin C mesotherapy with diode laser ablation therapy for the management of physiologic gingival hyperpigmentation in terms of clinical outcome, pain perception and patients’ satisfaction.

## Methods

### Study design

A randomized, parallel double-blinded clinical trial was carried out in accordance with the modified Helsinki’s code for human clinical studies 2013 [24], and the CONSORT guidelines for reporting randomized clinical trials 2010 [25].

This clinical trial received approval from the Research Ethics Committee at the Faculty of Dentistry, Alexandria University (IRB 00010556)-(IORG 0008839), and was registered on ClinicalTrials.gov (NCT05608057) on (01/11/2022). The study protocol was thoroughly explained to each participant, and a written informed consent was obtained.

### Study setting and patient selection

Patients were recruited from the outpatient clinic of the Department of Oral Medicine, Periodontology, Oral Diagnosis and Oral Radiology, Faculty of Dentistry, Alexandria University. After conducting an initial assessment that included reviewing medical and dental histories and performing clinical examinations to determine subject eligibility, 26 subjects met the following inclusion criteria: adults of either sex between 18 and 40 years old with physiologic melanin pigmentation in the anterior esthetic zone of the maxillary or mandibular gingiva, and who were free from any systemic diseases or conditions.

On the other hand, the exclusion criteria included: smokers, pregnant and lactating women, drugs intake which associate with gingival melanin pigmentation such as antimalarials drugs, phenothiazines and oral contraceptives, patients with periodontal disease, and patients with known hypersensitivity to ascorbic acid.

### Study sample and sample size estimation

Sample size was estimated assuming 5% alpha error and 80% study power. The mean (SD) Pigmentation score after 3 months is assumed to be 0.90 (0.55) for the Vitamin C Mesotherapy [26] and 0.2 (0.41) for the Diode Laser [27]. Based on the difference between independent means using the highest SD = 0.55 to ensure enough power. The minimum sample size was calculated to be 12 patients per group, increased to 13 patients to make up for lost to follow up. Total sample = number per group x number of groups = 13 × 2 = 26 patients. Sample size was based on Rosner’s method [28] calculated by G*Power 3.1.9.7 [29].

### Randomization, allocation concealment and blinding

Participants were randomly allocated into two groups using computer generated random list. The lists were placed in opaque, sealed envelopes, and organized in sequential manner by a dental assistant who was not involved in the study. Subsequently, each envelope was opened at the time of intervention [30].

This study was double-blinded, where both the evaluator of the outcomes and the statistician were blinded to the type of intervention used. Given the differences in the intervention techniques utilized in the two groups, blinding of both the operator and the patient could not be possibly achieved.

### Patient preparation

Pre-operative preparation phase was performed which included phase I therapy; this involved professional scaling using ultrasonic scalers and comprehensive oral hygiene instructions for all patients ensuring optimal oral health conditions prior to any procedure.

### Operative procedures

Eligible participants were randomly allocated into test group (G1. Mesotherapy) and control group (G2. Diode laser). All interventions were performed by the same operator (S.E.), thereby minimizing any potential variations in operator skills.

In both groups, infiltration anesthesia was administered at the target area using a local anesthetic agent; Alexandricaine 1:100000 (1.7 ml Articaine hydrochloride with 1:100000 Adrenaline, Alexandria Co. for Pharmaceuticals & Chemical Industries, Alexandria, Egypt).

 Group 1 [Vitamin C intra-mucosal injection (G1. Mesotherapy)] received intra-mucosal field injections of 1–1.5 ml Cevarol (L-Ascorbic acid 1000 mg/5 ml, Memphis Pharmaceuticals & Chemical Industries, Cairo, Egypt) using insulin syringe (30 Gauge 1 cc 0.33 mm × 8 mm 5/16″ needle) [14, 15].

**Fig. 1:**
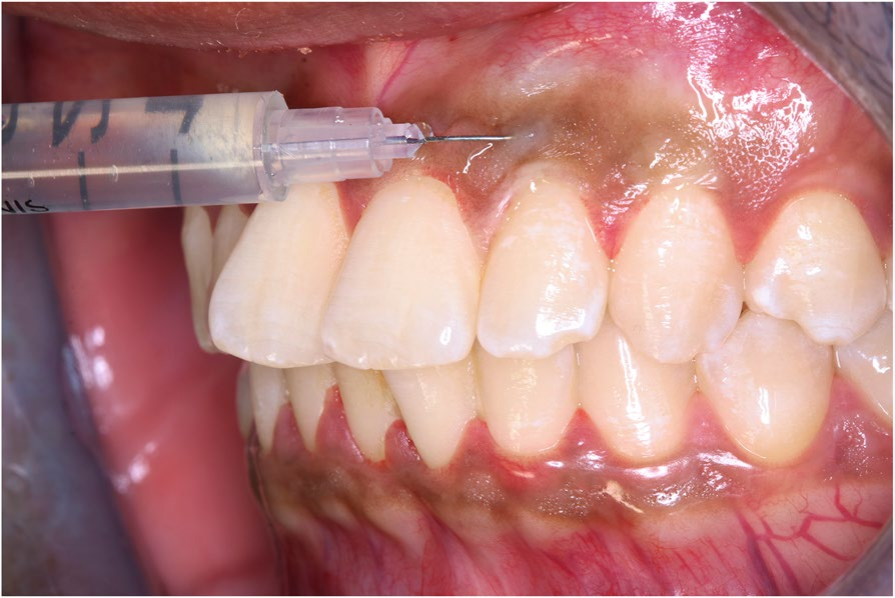
Illustration of Vitamin C intramucosal injection (Mesotherapy technique)

The intra-mucosal injection procedure involved inserting the needle just beneath the surface of the gingival epithelium, aligning it parallel to the junction between the epithelium & connective tissue. While ensuring that the bevel of the needle faced upward, an injection volume of 0.1 ml was administered at each point until the tissues blanched. This process was repeated across the entire pigmented area, while maintaining a distance of 2 to 3 mm between injection sites (Fig. 1).

Each patient underwent four treatment sessions, with a one-week interval between each session. Follow-up evaluations were conducted during each visit, and gingival depigmentation was assessed at 1 month, 2 months, and 3 months following the completion of the treatment procedure.

 Group 2 [Diode laser ablation (G2. Diode Laser)] was managed using 980 nm Diode laser (MEDENCY Primo Dental Laser, Italy). A comprehensive overview of the laser parameters can be found in (Table 1).

**Table 1:** Laser parameters

Laser wavelength	980 nm
Continuous wave/ pulsed mode	Continuous wave mode
Power	1.5 W
Contact/non-contact mode	Contact mode
Fiber-optic tip diameter	300 μm

To prevent overheating of the tissues, the tip was moved using small brush strokes in a back and forth motion. Throughout the procedure, the fiber tip was regularly cleaned using gauze soaked in saline solution. To ensure the preservation of the delicate gingival margin and interdental papilla, and to minimize the risk of gingival recession, the elimination of gingival hyperpigmentation was performed with a 1 mm distance maintained from the free gingiva. Epithelial remnants were cleared using gauze soaked in normal saline, serving to enhance visualization and to provide a cooling effect on the tissues. The ablation process was concluded when a whitish pink color was attained throughout the treated area.

### Post-operative care

All patients were given the following postoperative instructions: to refrain from consuming hot, spicy, hard, or rough foods for the first 24-hours, and to avoid any actions that may cause trauma to the treated area during the healing process. No medications were prescribed.

### Assessment

Clinical assessment was conducted at multiple time points, including the baseline examination and follow-up evaluations at 1, 2, & 3 months after completion of the treatment.

**Fig. 2:**
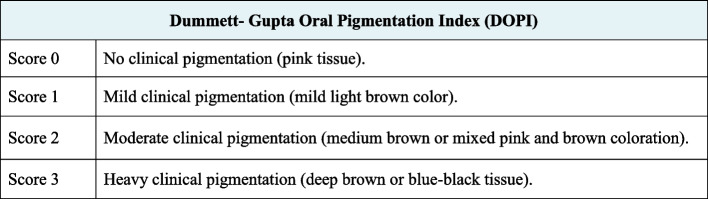
Dummet-Gupta Oral Pigmentation Index (DOPI)

**Fig. 3:**
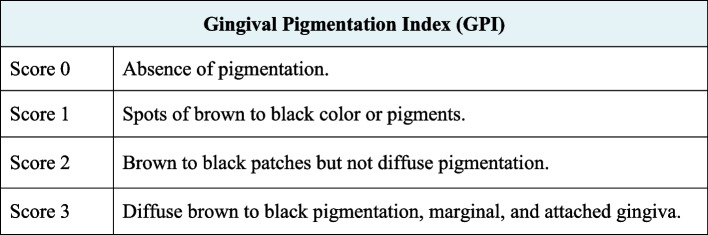
Gingival Pigmentation Index (GPI)

**Fig. 4:**
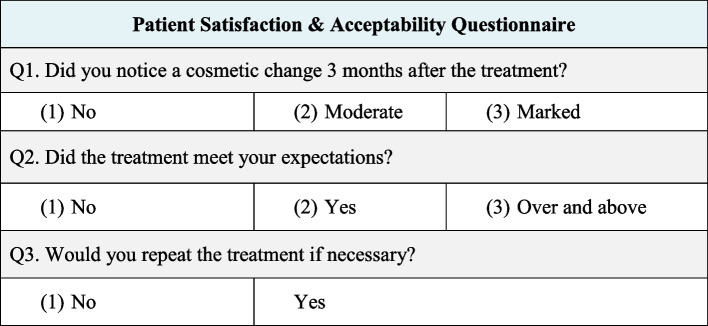
Satisfaction & Acceptability Questionnaire

The following parameters were evaluated:

1. Dummett- Gupta Oral Pigmentation Index (DOPI): [31] to assess the degree/intensity of gingival pigmentation, scored as in (Fig. 2).
2. Gingival Pigmentation Index (GPI): [32] to assess the area/ distribution of gingival pigmentation, scored as in (Fig. 3).
3. Visual Analogue Scale (VAS): [33] A pain intensity scale ranging from 0 to 10 was employed to assess and record the level of pain experienced by each patient. Following the treatment, patients were instructed to indicate their perception of pain immediately after the procedure, as well as on the 1st and 7th postoperative days.
4. Patient satisfaction and acceptability questionnaire: [14, 34] After 3 months (post-completion of the treatment), the participants were requested to provide answers to three questions aimed at evaluating their degree of contentment with each approach (Fig. 4).

### Statistical analysis

Data were analyzed using IBM SPSS version 25, Armonk, NY, USA. DOPI, GPI, and VAS were non-normally distributed and presented mainly using median, minimum, and maximum in addition to mean and standard deviation. Comparisons between groups was done using Mann Whitney U test with Bonferroni correction for multiple comparison adjustment while changes across time intervals were assessed using Friedman test followed by post hoc test with Bonferroni correction. Differences in patient satisfaction were assessed using Pearson Chi Square and Fisher’s Exact tests. Intra-examiner and inter-examiner reliability was calculated using Kappa Statistics. All tests were two tailed and the significance level was set at *p* value ≤0.05.

## Results

### Demographic data

A total of 26 participants were enrolled in the study and equally randomized into two groups: Group I, the Mesotherapy Group, and Group II, the Laser Group, with 13 participants in each group. Group I received intra-mucosal injection of vitamin C, while Group II was treated with the 980 nm diode laser. The mean age of participants in Group I was 23.00 years, while in Group II it was 21.62 years. The gender distribution in both groups was similar, with 15.4% males and 84.6% females. There was no statistically significant difference observed in terms of mean age or gender distribution between the two groups, as indicated in (Table 2).

**Table 2:** Demographic variables

		Group I (*n* = 13)	Group II (*n* = 13)	*P* value
Age: mean (SD)		23 (2.31)	21.62 (0.96)	0.063
Gender: n (%)	Males	2 (15.4%)	2 (15.4%)	1.00
	Females	11 (84.6%)	11 (84.6%)	

Statistically significant difference at *p* value≤0.05

**Fig. 5:**
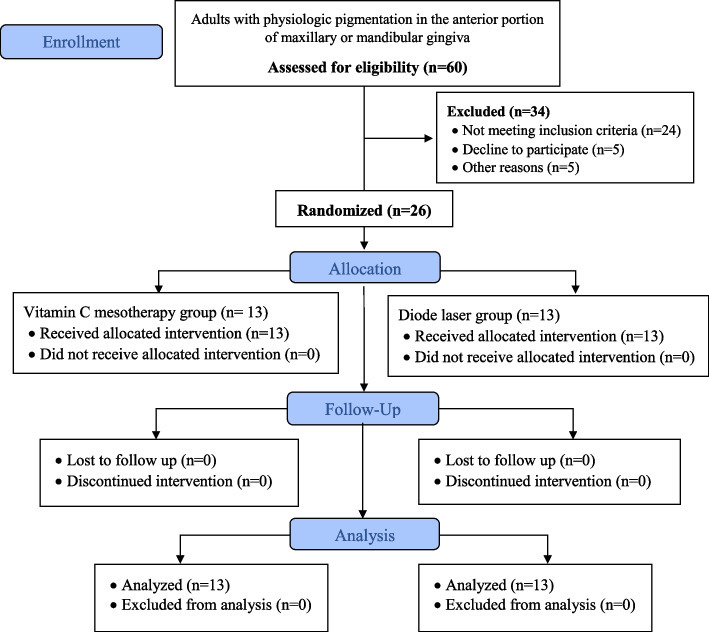
CONSORT flowchart of the study

All eligible participants included in the study adhered to the treatment protocol without any instances of dropouts or significant adverse effects, as illustrated in the study flowchart. (Fig. 5).

### Inter- and intra-examiner reliability

The inter- & intra-examiner reliability for DOPI & GPI scores were assessed using Kappa statistics, and the results ranged from 0.90 to 1.00 indicating an excellent level of agreement between examiners, and for repeated measurements by the same examiner across time.

### Dummett-Gupta Oral pigmentation index (DOPI) (Table 3)

#### Comparison within group (intragroup comparison)

In terms of intragroup comparison, the Mesotherapy group showed a significant reduction in DOPI scores across time (*P* = 0.043), with a significant difference observed between baseline & follow-up visits at 1, 2, and 3 months. Similarly, the Laser group also showed a significant improvement in DOPI scores from baseline to follow-up visits (*P* < 0.0001).

**Table 3:** Intergroup and intragroup comparison of DOPI scores at different time intervals

		Group I (n = 13)	Group II (*n* = 13)	*P* value
Baseline	Mean (SD)	2.46 (0.42)	2.26 (0.51)	0.434
	Median (IQR)	2.60 (0.80)	2.00 (0.82)	
	Min – Max	1.80–3.00	1.60–3.00	
1 month after completion of Tx	Mean (SD)	1.13 (0.35)	0.27 (0.36)	< 0.0001*
	Median (IQR)	1.00 (0.59)	0.16 (0.55)	
	Min – Max	0.67–1.67	0.00–1.00	
2 months after completion of Tx	Mean (SD)	0.95 (0.35)	0.23 (0.34)	< 0.0001*
	Median (IQR)	1.00 (0.63)	0.00 (0.400)	
	Min – Max	0.30–1.50	0.00–1.00	
3 months after completion of Tx	Mean (SD)	0.95 (0.35)	0.23 (0.35)	< 0.0001*
	Median (IQR)	1.00 (0.63)	0.00 (0.45)	
	Min – Max	0.30–1.50	0.00–1.00	
*P* value		< 0.0001*	< 0.0001*	

*Statistically significant difference at *p* value≤0.05

However, no significant difference was detected in DOPI scores between the 1, 2, and 3 months follow-up visits in either group.

#### Comparison between the two groups (intergroup comparison)

At baseline, there was no statistically significant difference in pigmentation intensity between the two groups (*P* = 0.434). However, at 1, 2, & 3 months of follow-up, there was a significant difference in pigmentation intensity between Mesotherapy group & Laser group (*P* < 0.0001), with the Laser group showing greater improvement in pigmentation intensity reduction.

### Gingival Pigmentation Index (GPI) (Table 4)

#### Comparison within group (intragroup comparison)

In the Mesotherapy group, there was a statistically significant reduction (*P* < 0.0001) in GPI scores across different time intervals. Similarly, the Laser group demonstrated a statistically significant reduction in GPI scores at baseline and at follow up visits (*P* < 0.0001).

**Table 4:** Intergroup and intragroup comparison of GPI scores at different time intervals

		Group I (*n* = 13)	Group II (*n* = 13)	*P* value
Baseline	Mean (SD)	2.54 (0.52)	2.69 (0.480)	0.511
	Median (IQR)	3.00 (1)	3.00 (1)	
	Min – Max	2–3	2–3	
1 month after completion of Tx	Mean (SD)	1.92 (0.64)	0.62 (0.65)	< 0.0001*
	Median (IQR)	2.00 (1)	1.00 (1)	
	Min - Max	1–3	0–2	
2 months after completion of Tx	Mean (SD)	1.92 (0.64)	0.54 (0.66)	< 0.0001*
	Median (IQR)	2.00 (1)	0.00 (1)	
	Min – Max	1–3	0–2	
3 months after completion of Tx	Mean (SD)	1.92 (0.64)	0.54 (0.66)	< 0.0001*
	Median (IQR)	2.00 (1)	0.00 (1)	
	Min - Max	1–3	0–2	
P value		< 0.0001*	< 0.0001*	

*Statistically significant difference at *p* value≤0.05

Nevertheless, there was no statistically significant difference between the follow up visits scores at 1, 2 and 3 months in both groups.

#### Comparison between the two groups (intergroup comparison)

At Baseline, no significant difference was found in preoperative distribution of pigmentation area between the two groups (*P* = 0.511).

**Fig. 6:**
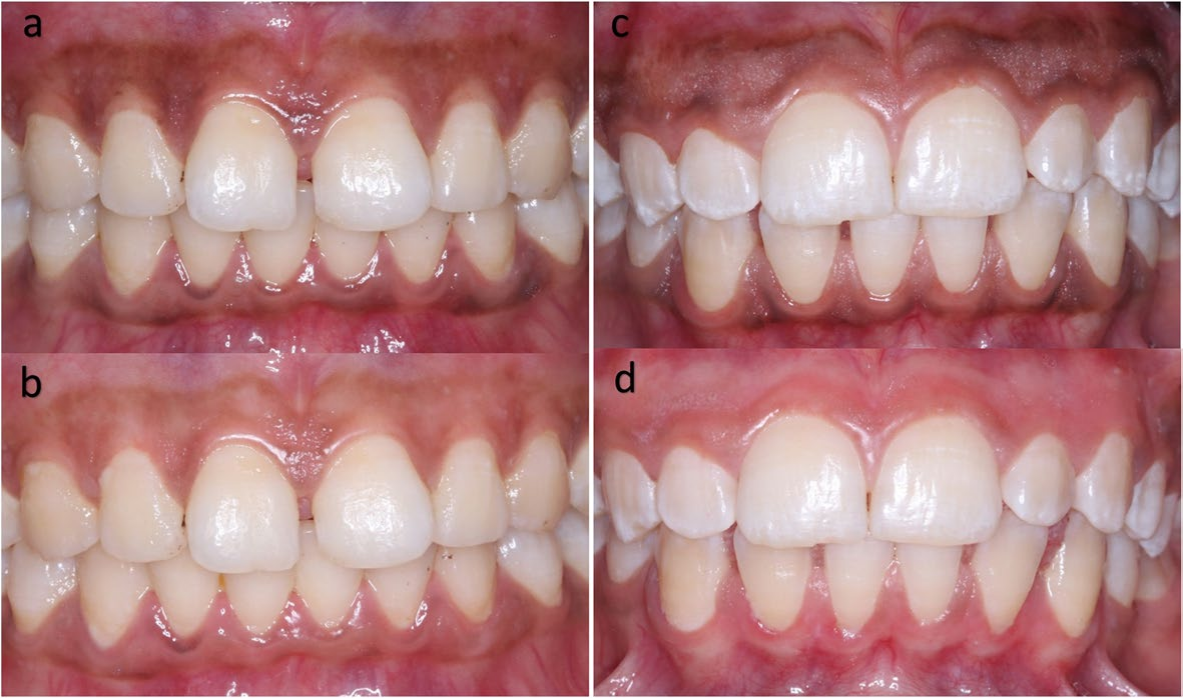
Intraoral photographs of a patient from Group 1 at baseline (**a**), and 3 months after completion of treatment (**b**) using vitamin C mesotherapy technique, compared with photographs of a patient from Group 2 at baseline (**c**), and after 3 months (**d**) following diode laser ablation therapy

However, at 1, 2 & 3 months follow up, there was a statistically significant difference (*P* < 0.0001) in area of pigmentation value in favor of the Laser group (Fig. 6) here.

### Visual analogue scale (VAS): (intergroup comparison)

**Fig. 7:**
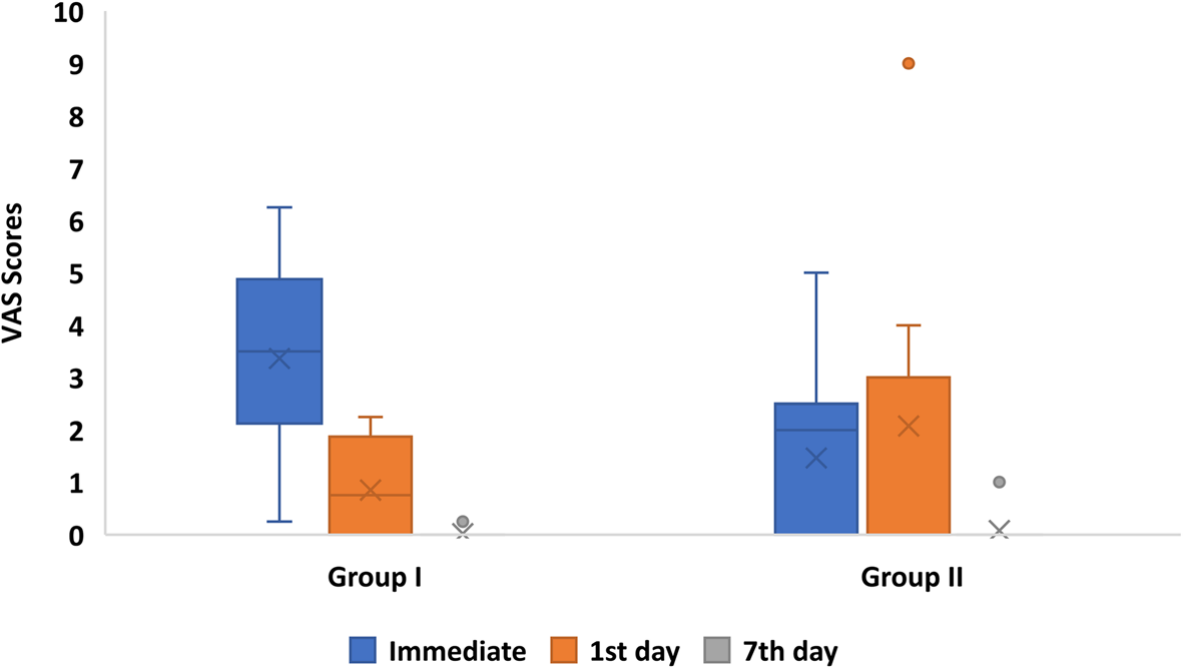
Comparison of Visual Analogue Scale (VAS) between groups immediately postoperative, at 1st and 7th days postoperatively

A statistically significant difference (*P* = 0.005) was observed in immediate postoperative pain scores between the two groups, favoring the Laser group. However, no significant differences in pain scores on the 1st and 7th postoperative days were detected between the two groups (Fig. 7).

### Patient satisfaction and acceptability questionnaire

**Fig. 8:**
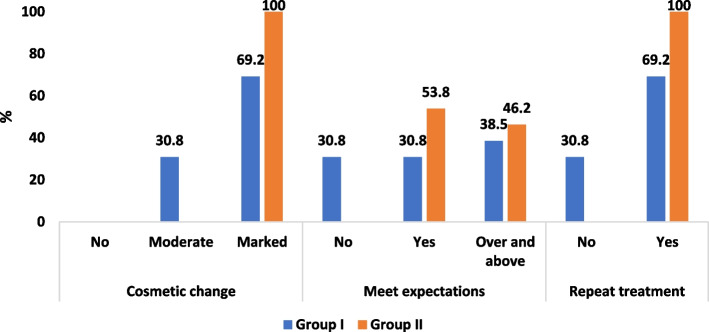
Satisfaction Questionnaire

The study’s results revealed no notable difference in patients’ satisfaction and acceptance of the treatment method between the two groups, as demonstrated in (Fig. 8).

## Discussion

The role of pink (gingival) esthetics in enhancing the overall appearance, self-confidence, and personality of an individual cannot be overstated. Recent advances in esthetic dentistry aim to harmonize the functional and esthetic aspects of a smile, while taking into consideration patients’ unique values and needs. Therefore, there is a need to investigate cost-effective yet efficient techniques to ensure the accessibility of these esthetic treatments for all those who desire them.

A variety of treatment modalities have been proposed and presented in the literature for the management of gingival hyperpigmentation, with lasers being the prevailing choice in contemporary practice. In spite of the overall advantages of laser depigmentation, it remains a technique sensitive procedure that requires expensive equipment and proper training before usage. Conversely, while vitamin C has gained widespread recognition as a depigmenting agent in dermatology, its application for gingival depigmentation has been sparsely documented [18]. The minimally invasive oral mesotherapy technique enables targeted delivery of vitamin C directly to the affected area, while being a cost-effective modality of treatment [23].

Hence, this study was conducted to evaluate and compare the clinical performance, pain perception and patient satisfaction of vitamin C mesotherapy and diode laser ablation therapy in managing physiologic gingival hyperpigmentation. To the best of our knowledge, this is the first randomized clinical trial to compare between these two treatment modalities.

Our findings indicate that both treatment modalities were successful in effectively managing gingival hyperpigmentation, highlighting the efficacy of both approaches in managing this common esthetic concern.

In the diode laser group, there was a significant reduction in pigmentation indices following treatment. These results were consistent with several other published studies [10, 27, 34–37]. According to a systematic review and meta-analysis investigating the optimal method for managing physiologic gingival hyperpigmentation, lasers, particularly diode lasers, were found to be the most commonly employed technique demonstrating superior esthetic results, minimal pain, accelerated healing, and high levels of patients’ preference and satisfaction post-treatment [38].

With laser depigmentation, the absorption of the laser light by melanocytes is influenced by the wavelength of the laser used, and its depth of penetration. Melanin, being the target chromophore, has an absorption spectrum ranging between 351 and 1064 nm. Hence, 980 nm diode laser was chosen as it is highly absorbed by soft tissue with a particular affinity for chromophores, like melanin and oxyhemoglobin. Additionally, it offers great tissue penetration, reaching depths of up to 10 mm [10]. The efficient absorption of laser energy by melanin, combined with the tissue penetration capacity make the diode laser an ideal option for ablation of melanin pigments present within the gingival tissue within a relatively short procedure time.

In the vitamin C mesotherapy group, the pigmentation indices (DOPI and GPI) showed a decrease in indices’ scores with a significant statistical difference between baseline scores and follow-up visits’ scores, confirming the effectiveness of vitamin C as a depigmenting agent. These findings aligned with other clinical studies that revealed significant improvement in gingival color with the vitamin C mesotherapy technique [14, 15, 20–22]. On the other hand, no statistically significant difference was detected upon comparing post-operative follow-up visits. This observation is consistent with the studies conducted by Yussif et al. (2016) and Dawar et al. (2022) [20, 22]. This may be explained by the narrow range of pigmentation indices which poses a challenge in assessing the slow progress during follow-up visits.

There are various mechanisms by which vitamin C reduces the gingival hyperpigmentation. Firstly, it suppresses the tyrosinase activity, the rate-limiting enzyme in melanin biosynthesis, through cytoplasmic acidification. Given that vitamin C is an acidic compound, it causes intracellular acidification of melanocytes, which reduces melanin production since the activity of tyrosinase is generally minimal in an acidic environment [39]. Moreover, vitamin C competes with tyrosinase for binding to copper ions, which are essential co-factors for the activity of tyrosinase. By binding to copper ions, vitamin C prevents their interaction with tyrosinase, and further hinders melanin synthesis [40]. Secondly, vitamin C exhibits strong antioxidant properties, thus scavenging free radicals, which are known to stimulate melanogenesis [41]. Thirdly, vitamin C acts as a promoter of collagen biosynthesis, [42] preventing its breakdown which reduces tissue damage, enhances proper keratinocytes differentiation, and indirectly reduces melanin production [43, 44]. Considering this, vitamin C may also present a better treatment option than surgical depigmentation techniques for individuals with thin gingival biotype.

As a result of the aforementioned mechanisms, vitamin C has a significant impact on hyperpigmentation, although its effects may take longer to manifest compared to the rapid outcomes achieved through laser therapy.

Furthermore, previous studies have demonstrated the synergistic effects of vitamin C injection on the enhancement of the overall gingival health and reduction of inflammation [45, 46]. As such, the utilization of vitamin C as a depigmenting agent, not only fulfills its intended purpose, but also significantly enhances the overall health of the gingival tissues. This combined influence undeniably fortifies and optimizes the esthetic outcome of the treatment procedure.

Although the two treatment modalities have proven to be statistically significant in reducing the intensity and areas of gingival pigmentation, the intergroup comparison showed statistically significant difference in favor of the Laser group.

This could be attributed to the fact that the mechanism of gingival depigmentation by diode laser and vitamin C is entirely distinct. As diode laser therapy involves the complete removal of the surface epithelium, along with the melanocytes contained within it [47], while vitamin C works by interrupting the process of melanogenesis, as previously stated.

Hence, we may conclude that the choice of the gingival depigmentation technique should be driven by multiple factors; including the patient’s financial status, the patient’s compliance to treatment, the operator’s skills, the overall health of the gingival tissue, the intensity of the gingival pigments and the gingival biotype.

In terms of pain perception, the participants in the Laser group reported less levels of pain immediately after treatment than those in the Mesotherapy group. This could be attributed to several factors. The pain scores reported just after treatment in the Mesotherapy group were probably only due to the discomfort associated with the multiple injections required for that treatment method. In contrast, the reduction in pain reported by laser group could be explained by the protein coagulum that forms on the treated surface, acting as a biologic barrier that seals sensory nerves endings. Additionally, the photobiomodulation effects of laser therapy can play a role in reducing pain [10].

In our study, a questionnaire was used to evaluate the patients’ satisfaction with the esthetic outcome achieved after 3 months, as well as their acceptability of the treatment method employed in both groups. Overall, the patients expressed high levels of satisfaction, indicating positive esthetic outcomes for both treatment modalities. Notably, there was no statistically significant difference observed between the two groups in terms of satisfaction and acceptability of the treatment method.

While the present study provides valuable insights into the research topic, there are some limitations that should be taken into consideration. Longer follow up periods may be required to assess the recurrence rate following each depigmentation technique. Besides, the mesotherapy technique involves multiple injections, which can be uncomfortable for some patients. This can limit the use of this technique in certain patient populations, such as those with needle phobia. In addition, the requirement for multiple visits for vitamin C mesotherapy can pose a drawback as it increases the need for patient’s compliance.

Another consideration involves the random treatment assignment of both maxillary and mandibular gingiva to either vitamin C mesotherapy or diode laser ablation, which could be seen as a limitation, as the maxillary gingiva has a relatively thick phenotype compared to that of the mandibular region. Therefore, it is advised that future studies take into consideration the gingival phenotype and consider its potential influence on the outcomes.

## Conclusion

Based on the favorable outcomes reported in this study, it could be concluded that vitamin C mesotherapy is an effective and safe approach for managing gingival hyperpigmentation. Although diode laser yields better and earlier results, vitamin C mesotherapy offers a minimally invasive and applicable treatment option that is both cost-effective and esthetically satisfying for the patients.

## Data Availability

All data included in this study are available from the corresponding author upon request.

## Abbreviations

DOPI: Dummett-Gupta Oral Pigmentation Index
GPI: Gingival Pigmentation Index
VAS: Visual Analogue Scale
Tx: Treatment
